# Blood pressure components and incident cardiovascular disease and mortality events among Iranian adults with chronic kidney disease during over a decade long follow-up: a prospective cohort study

**DOI:** 10.1186/s12967-018-1603-7

**Published:** 2018-08-15

**Authors:** Ashkan Hashemi, Sormeh Nourbakhsh, Samaneh Asgari, Mohammadhassan Mirbolouk, Fereidoun Azizi, Farzad Hadaegh

**Affiliations:** 1 Prevention of Metabolic Disorders Research Center, Research Institute for Endocrine Sciences, Shahid Beheshti University of Medical Sciences, No. 24, Parvaneh Street, Velenjak, P.O. Box: 19395-4763, Tehran, Iran; 2 Johns Hopkins Ciccarone Center for the Prevention of Heart Disease, Johns Hopkins Hospital, Baltimore, USA; 3 Endocrine Research Center, Research Institute for Endocrine Sciences, Shahid Beheshti University of Medical Sciences, Tehran, Iran

**Keywords:** Blood pressure, Systolic blood pressure, Diastolic blood pressure, Pulse pressure, Chronic kidney disease, Cardiovascular disease

## Abstract

**Background:**

To explore the association between systolic and diastolic blood pressure (SBP and DBP respectively) and pulse pressure (PP) with cardiovascular disease (CVD) and mortality events among Iranian patients with prevalent CKD.

**Methods:**

Patients [n = 1448, mean age: 60.9 (9.9) years] defined as those with estimated glomerular filtration rate < 60 ml/min/1.73 m^2^, were followed from 31 January 1999 to 20 March 2014. Multivariable Cox proportional hazard models were applied to examine the associations between different components of BP with outcomes.

**Results:**

During a median follow-up of 13.9 years, 305 all-cause mortality and 317 (100 fatal) CVD events (among those free from CVD, n = 1232) occurred. For CVD and CV-mortality, SBP and PP showed a linear relationship, while a U-shaped relationship for DBP was observed with all outcomes. Considering 120 ≤ SBP < 130 as reference, SBP ≥ 140 mmHg was associated with the highest hazard ratio (HR) for CVD [1.68 (1.2–2.34)], all-cause [1.72 (1.19–2.48)], and CV-mortality events [2.21 (1.16–4.22)]. Regarding DBP, compared with 80 ≤ DBP < 85 as reference, the level of ≥ 85 mmHg increased risk of CVD and all-cause mortality events; furthermore, DBP < 80 mmHg was associated with significant HR for CVD events [1.55 (1.08–2.24)], all-cause [1.68 (1.13–2.5)] and CV-mortality events [3.0 (1.17–7.7)]. Considering PP, the highest HR was seen in participants in the 4th quartile for all outcomes of interest; HRs for CVD events [1.92 (1.33–2.78)], all-cause [1.71 (1.11–2.63)] and CV-mortality events [2.22 (1.06–4.64)].

**Conclusions:**

Among patients with CKD, the lowest risk of all-cause and CV-mortality as well as incident CVD was observed in those with SBP < 140, 80 ≤ DBP < 85 and PP < 64 mmHg.

## Background

Cardiovascular disease (CVD) is the major cause of morbidity and mortality among patients with chronic kidney disease (CKD) [1]. Poorly controlled hypertension is associated with increased risk of cardiovascular morbidity and mortality as well as higher risk and accelerated rate of kidney function deterioration in patients with CKD [2]. Thus, optimal BP control is vital in CKD patient management. However, the BP threshold for initiation and goal of treatment remains controversial due to conflicting evidence available [3]. Due to the inconsistency in the evidence supporting the idea of *‘‘the lower the better strategy’’*, the Joint National Committee (JNC) raised the BP goal for CKD patients from below 130/80 mmHg in JNC 7 [4] to a more liberal target of less than 140/90 mmHg in JNC 8 [5]. On the other hand, the latest report of The American College of Cardiology/American Heart Association (ACC/AHA) guideline for Prevention, Detection, Evaluation and Management of High Blood Pressure in Adults, again decreased the goal of BP lowering therapy among hypertensive CKD patients to below 130/80 mmHg [6].

The exact relationship between the components of blood pressure [SBP, DBP and their difference called pulse pressure (PP)] with CVD and all-cause mortality among CKD population, has not been consistent among studies. While some studies suggest for a linear relationship [7] or advocate for *‘‘the lower the better strategy’’* [8, 9], others report a J or U shaped association [10–12], depending on the specific BP components and type of outcomes studied. Among patients with incident CKD, Kovesdy et al. [13] indicated a linear association between SBP with CVD events and a U shaped relationship for both SBP and DBP with all-cause mortality. Interestingly, while Palit et al. [14] identified a strong association between higher PP and CVD events, they could not establish such a relationship between either SBP or DBP with mortality among patients with advanced CKD.

Since the studies mentioned above have mainly been conducted on Western populations, their results may not be applicable to other ethnicities such as Middle Eastern populations which have high incidence of CKD and its related risk factors such as hypertension and type 2 diabetes [15–18]. In the current study we have examined the association between different components of blood pressure (SBP, DBP, and PP) with CVD and mortality events in a long term population based study among an adult Tehranian population with prevalent CKD.

## Methods

### Patients and study design

“Tehran Lipid and Glucose Study” (TLGS) is a dynamic prospective longitudinal population-based study, being performed on a representative sample of Tehran, the capital city of Iran. The aim of the study is to determine the prevalence of non-communicable disease risk factors. TLGS enrollment was in two phases: First phase (1999–2001) and the second phase (2001–2005). Data collection is ongoing and scheduled to continue for at least 20 years, at 3-year intervals, details of the design and enrollment of the TLGS cohort have been reported previously [19].

From a total of 9731 participants, aged ≥ 30 years, (8064 individuals from phase I and 1667 new participants from phase 2), there were only 1761 participants with prevalent CKD (estimated glomerular filtration rate; eGFR < 60 ml/min/1.73 m^2^) in the cross sectional phases of TLGS. We excluded those with missing data on fasting plasma glucose (FPG), standard 2-h post challenge plasma glucose (2 h-PCG), total cholesterol (TC), body mass index (BMI), smoking habits and eGFR at baseline (n = 125), and those with no follow-up (n = 188), leaving 1448 CKD patients, who were followed until 20 March 2014. Furthermore, when we focused on CVD and its mortality as outcome, those with prevalent CVD (n = 216) were also excluded, leaving 1232 individuals.

Written informed consent was obtained from all participants and the medical ethics committee of the Research Institute for Endocrine Sciences approved the study proposal.

### Clinical and laboratory measurements

Information, collected by a trained interviewer using a standardized questionnaire, which included demographic characteristics, smoking status, medication regimen (antihypertensive, lipid-lowering and anti-diabetic agents) and past medical history of CVD. Details of anthropometric measurements are discussed elsewhere [19]. BMI was calculated as weight in kilograms divided by square of height in meters. Using the MONICA protocol [20], trained personnel obtained two measurements of SBP and DBP on the right arm of participants after they rested in a sitting position for 15 min, using a standardized mercury sphygmomanometer (calibrated by the Iranian Institute of Standards and Industrial Researches). The 1st and 5th Korotkoff sounds were considered as SBP and DBP respectively; BP for each patient were measured twice at least 30 s apart, and the average of the two were reported and used for analysis in this study [20, 21].

We measured FPG, standard 2 h-PCG, TC and serum creatinine (Cr) using blood samples, drawn from subjects after 12–14 h of overnight fasting. All sampling was done between 7:00 and 9:00 AM and analyzed on the same day in the TLGS research laboratory, using commercial kits (Pars Azmoon Inc., Tehran, Iran) by a Selectra 2 auto analyzer (Vital Scientific, Spankeren, The Netherlands); serum Cr level was assessed by the Jaffe kinetic colorimetric method. According to manufacturer’s recommendation, reference intervals were 53–97 mmol/l (0.6–1.1 mg/dl) in women and 80–115 mmol/l (0.9–1.3 mg/dl) in men; the sensitivity of the assay was 0.2 mg/dl. In baseline and follow-up phases both intra and inter-assay CVs were less than 3.1%. Using lyophilized serum controls in normal and abnormal ranges, assay performance was monitored after every 25 tests. All samples were assayed only when internal quality control met the standard criteria [19, 22].

### Outcome measurements

Details of cardiovascular data collection can be found elsewhere [19]. To summarize, the study participants were annually followed. Those who were not available on the primary call were contacted again (up to 4 times a year) and if they did not respond, their data were considered as missing. A trained nurse asked the subjects regarding any medical incidents and later a trained physician collected complementary data on each of those incidents by gathering information from their medical files or during home visits. Hospital records or death certificates were used for mortality event records. An outcome committee, including a principal investigator, a cardiologist, an endocrinologist, an epidemiologist and the physician who collected outcome data, was formed to evaluate the results and other experts were invited as needed. Clinical conditions were assessed using the 10th revision of the International Classification of Diseases (ICD-10) and American Heart Association classification for cardiovascular events. Outcomes of interest were all-cause mortality and the first CVD events which included: Definite myocardial infarction (with positive ECG and cardiac biomarkers), probable myocardial infarction (positive ECG and cardiac signs/symptoms with negative or equivocal biomarkers), unstable angina (new cardiac symptoms or changing symptoms patterns and positive ECG findings with normal biomarkers), angiographic approved coronary heart disease and CVD related death.

### Definition of terms

The “*Chronic Kidney Disease Epidemiology Collaboration (CKD*-*EPI)”* formula, was used for calculating eGFR (ml/min per 1.73 m^2^). CKD-EPI equation, as follows:

$$eGFR \, = \, 141 \times min \, \left( {Serum \, creatinine/\kappa , \, 1} \right)^{\alpha } \times \, max \, \left( {Serum \, creatinine/\kappa , \, 1} \right)^{ - 1.209} \times \, 0.993^{Age} \times \, 1.018 \, \left[ {if \, female} \right]$$

In this formula eGFR is expressed in ml/min per 1.73 m^2^; serum creatinine is expressed in mg/dl, κ is 0.7 for females and 0.9 for males, α is − 0.329 for females and − 0.411 for males; min indicates the minimum of serum creatinine/κ or 1, and max indicates the maximum of serum creatinine/κ or 1 [23]. Based on the Kidney Disease Outcome Quality Initiative guidelines, CKD is defined as either kidney damage or eGFR < 60 ml/min per 1.73 m^2^ for > 3 months [24].

Regarding smoking status, participants were placed into three groups, never, former and current smokers, based on their response to the questionnaire. Current smoker refers to an individual who uses any tobacco product (cigarettes, pipe or water pipe) on a daily or occasional basis. Type 2 diabetes (T2D) was defined according to the American Diabetes Association with FPG levels ≥ 126 mg/dl (7 mmol/l) or 2 h-PCPG ≥ 200 mg/dl (1.1 mmol/l) or usage of any anti-diabetic medication [25]. Hypercholesterolemia was defined by serum total cholesterol ≥ 200 mg/dl of (≥ 5.17 mmol/L) or receiving lipid lowering agents. PP was calculated by subtracting the DBP from SBP. A physician diagnosed CVD, prior to entering the study, was considered as prevalent CVD.

### Statistical analysis

Mean (SD) values for continuous variables and frequency (%) for categorical ones of baseline characteristics are presented. Comparisons of baseline characteristics between dead and alive participants were conducted using Student’s t-test for continuous and the Chi square test for categorical variables.

Follow up duration was considered the time between entrance to the study and the end points; end points were measured as CVD and mortality events. Also, censored data was considered as subjects with loss to follow-up, or having left the residential area, non-CVD mortality (for CV-mortality endpoint) event or until end of follow up (i.e. 20 March 2014), whichever occurred earlier.

Multivariable Cox proportional hazard models (age adjusted as time scale) were used to evaluate associations of blood pressure components for CVD, CVD and total mortality. In this analysis SBP and DBP were examined separately as categorical variables (SBP: ≤ 120, 120–130 (as reference), 130–140, and ≥ 140 mmHg; DBP: ≤ 80, 80–85 (as reference) 85–90 and ≥ 90 mmHg). Quartiles of PP were also considered for our data analysis, considering the first quartile as reference.

Adjustment for age was done using age as the time scale [26]. Associations between BP components and different outcomes were evaluated in two models: Model 1, included gender; Model 2 was further adjusted for both potential confounders including BMI, T2D, hypercholesterolemia, eGFR, smoking status (never smoker as reference) and anti-hypertensive medication (only for total population) and also for prevalent CVD for all-cause mortality. We found no significant p-values (minimum > 0.2) for interactions between different blood pressure components (SBP, DBP and PP) and gender for either CVD or total mortality; hence, we adjusted for gender, to reach full statistical power. Similarly, we also found no interaction between prevalent CVD and blood pressure components for total mortality (all p-values > 0.4). The analysis was also stratified based on the consumption of anti-hypertensive medications at baseline for all outcomes, excluding CV mortality. The fractional polynomial method (FP) was used to check the dose-response association between SBP, DBP and PP with CVD, all-cause and CV-mortality in a confounder adjusted model with three knots (at 25th, 50th and 75th percentiles) [27].

The Cox proportional hazard assumption was checked by the Schoenfield residual test and no violation was found. All analyses were done using Stata version 12 (Stata Corp LP, Stata Statistical Software: Release 12, College Station, TX, USA) and a two-tailed p-values < 0.05 were considered significant.

## Results

The study population included 1448 patients with prevalent CKD. Mean (SD) for age, BMI and eGFR in total population were 60.9 (9.9) years, 28.2 (4.3) kg/m^2^ and 52.8 (6.3) ml/min/1.73 m^2^, respectively. The prevalence of T2D, hypercholesterolemia, current smoking and prevalent CVD was 26, 78.4, 8.8 and 14.9%, respectively. Furthermore, the prevalence of BP lowering medications among the study population was 27.6%. During the follow up 305 individuals died. Comparing the baseline characteristics of survivors versus non-survivors, the non-survivor group had a higher means of age (58.96 vs. 68.13 years), SBP (131.64 vs. 144.12 mmHg), FPG (107.5 vs. 128.83 mg/dl), as well as higher prevalence of T2D (20.6 vs. 46.2%), current smoking (8 vs. 12.1%) and prevalent CVD (12.5 vs. 23.9%), however this group had lower mean BMI (28.4 vs. 27.37 kg/m^2^) and total cholesterol (237.13 vs. 231.16 mg/dl) (Table 1).

**Table 1:** Baseline characteristics of the study population: Tehran Lipid and Glucose Study (1999–2014)

	Total (N = 1448)	Non-survivors (N = 305)	Survivors (N = 1143)	p-value
Age, years	60.9 (9.9)	68.13 (7.7)	58.96 (9.6)	< 0.001
BMI, kg/m^2^	28.2 (4.3)	27.37 (4.4)	28.4 (4.3)	< 0.001
SBP (mmHg)	134.27 (23.3)	144.12 (23.9)	131.64 (22.4)	< 0.001
DBP (mmHg)	81.4 (12.0)	82.3 (14.1)	81.23 (11.3)	0.22
FPG (mg/dl)	112.0 (47.19)	128.83 (61.6)	107.5 (41.4)	< 0.001
2 h-PCPG (mg/dl)	141.23 (71.9)	154.7 (87.7)	138.5 (67.9)	0.012
Total cholesterol (mg/dl)	235.9(48.4)	231.16(48.9)	237.13 (48.2)	0.06
eGFR, 60 ml/min/1.73 m^2^	52.8 (6.3)	50.3 (8.12)	53.45 (5.6)	< 0.001
Pulse pressure, (mmHg)	52.8 (18.3)	61.8 (18.7)	50.4 (17.4)	< 0.001
Hypertension medication, n (%)	399 (27.6)	115 (37.7)	284 (24.8)	< 0.001
Smoking status, n (%)				0.001
Never	1131 (78.1)	214 (70.2)	917 (80.2)	
Former	189 (13.1)	54 (17.7)	135 (11.8)	
Current	128 (8.8)	37 (12.1)	91 (8.0)	
Diabetes, n (%)	377 (26.0)	141 (46.2)	236 (20.6)	< 0.001
Hypercholesterolemia, n (%)	1135 (78.4)	231 (75.7)	904 (79.1)	0.21
Prevalent CVD, n (%)	216 (14.9)	73 (23.9)	143 (12.5)	< 0.001

SI conversion factors: To convert fasting plasma glucose and 2-h fasting plasma glucose concentrations to mmol/l, multiply by 0.05551; to convert total cholesterol values to mmol/l, multiply by 0.02586

Mean (SD), shown for continuous variables and p value was calculated by t-test; n (%), shown for categorical variables with p value according to chi-square test

*BMI* body mass index, *WC* waist circumference, *SBP* systolic blood pressure, *DBP* diastolic blood pressure, *FPG* fasting plasma glucose, *2h-PCPG* 2-h fasting plasma glucose

After a median follow-up of 13.9 years, among those free of CVD at baseline (n = 1232), 317 CVD events (n = 100, attributable to CV-mortality) occurred. Moreover among the whole population, including those with prevalent CVD (n = 1448), 305 all-cause mortality events occurred. The multivariate adjusted risk estimation of different systolic and diastolic blood pressures as well as PP quartiles for CVD and all-cause mortality events among the total population and those receiving anti-hypertensive medication, as well as untreated ones, are shown in Tables 2 and 3. Regarding CVD events, compared to the reference group, participants with SBP ≥ 140 mmHg had the highest HR for CVD events in the multivariable adjusted model, a pattern also seen in the untreated group; however, we found no such risk among the treated group [2.01 (0.89–4.57), p-value = 0.1]. Furthermore, in both the treated and untreated groups, SBP < 120 mmHg was not a significant predictor for CVD events [HR 1.39 (0.52–3.8) and 0.9 (0.59–1.36), respectively]. Focusing on DBP, in multivariate analysis, among untreated participants, those with DBP ≥ 85 mmHg (whether DBP 85–90 or ≥ 90 mmHg) had statistically significant risk. Moreover, pooling DBP 85–90 and ≥ 90 mmHg as a single group, DBP ≥ 85 mmHg showed significant risk for CVD among the total as well as treated and untreated populations; the corresponding multivariate adjusted HRs (CI) were 2.35 (1.08–2.26), 3.7 (1.75–7.7) and 1.95 (1.28–2.96), respectively.

**Table 2:** Multivariate adjusted risk estimation of different systolic and diastolic blood pressure as well as pulse pressure quartiles for Cardiovascular disease in the total population and those with and without anti-hypertensive medication: Tehran Lipid and Glucose study (1999–2014)

	BP category		Model 1				Model 2		
		E/N	HR		(95% CI)	p	HR	(95% CI)	P
Total (N = 1232)	SBP < 120	61/367	0.92		(0.63–1.34)	0.67	0.94	(0.64–1.38)	0.75
	120 ≤ SBP < 130*	49/224	Ref				Ref		
	130 ≤ SBP < 140	42/211	0.95		(0.63–1.44)	0.81	0.87	(0.57–1.32)	0.52
	SBP ≥ 140	165/430	1.67		(1.21–2.3)	0.002	1.68	(1.2–2.34)	0.002
Treated (N = 286)	SBP < 120	10/36	1.32		(0.5–3.5)	0.57	1.39	(0.51–3.8)	0.52
	120 ≤ SBP < 130*	7/27	Ref				Ref		
	130 ≤ SBP < 140	12/48	1.19		(0.46–3.04)	0.71	1.09	(0.42–2.87)	0.85
	SBP ≥ 140	66/175	1.69		(0.77–3.69)	0.19	2.01	(0.89–4.57)	0.1
Untreated (N = 946)	SBP < 120	51/331	0.87		(0.58–1.31)	0.51	0.9	(0.59–1.36)	0.61
	120 ≤ SBP < 130*	42/197	Ref				Ref		
	130 ≤ SBP < 140	30/163	0.85		(0.53–1.36)	0.5	0.8	(0.5–1.29)	0.37
	SBP ≥ 140	99/255	1.62		(1.12–2.34)	0.01	1.64	(1.13–2.39)	0.009
Total (N = 1232)	DBP < 80	132/544	1.61		(1.13–2.32)	0.009	1.55	(1.08–2.24)	0.02
	80 ≤ DBP < 85*	38/242	Ref				Ref		
	85 ≤ DBP < 90	45/165	1.88		(1.22–2.90)	0.004	1.93	(1.25–2.98)	0.003
	DBP ≥ 90	102/281	2.68		(1.85–3.89)	<0.001	2.63	(1.8–3.83)	<0.001
Treated (N = 286)	DBP < 80	24/77	2.13		(0.95–4.78)	0.065	2.00	(0.89–4.54)	0.09
	80 ≤ DBP < 85*	8/55	Ref				Ref		
	85 ≤ DBP < 90	11/38	2.1		(0.84–5.22)	0.11	1.96	(0.77–4.95)	0.16
	DBP ≥ 90	52/116	4.02		(1.9–4.85)	< 0.001	4.54	(2.13–9.67)	<0.001
Untreated (N = 946)	DBP < 80	108/467	1.5		(1.0–2.25)	0.049	1.43	(0.95–2.16)	0.09
	80 ≤ DBP < 85*	30/187	Ref				Ref		
	85 ≤ DBP < 90	34/127	1.84		(1.13–3.01)	0.015	1.94	(1.19–3.18)	0.008
	DBP ≥ 90	50/165	2.09		(1.33–3.29)	0.001	1.95	(1.23–3.08)	0.004
Total (N = 1232)	PP < 40*	46/327	Ref				Ref		
	40 ≤ PP < 50	70/291	1.5		(1.03–2.19)	0.034	1.44	(0.98–2.1)	0.06
	50 ≤ PP < 64	79/307	1.43		(0.98–2.08)	0.06	1.28	(0.88–1.87)	0.2
	PP ≥ 64	122/307	2.13		(1.48–3.06)	<0.001	1.92	(1.33–2.78)	0.001
Treated (N = 286)	PP < 40*	12/37	Ref				Ref		
	40 ≤ PP < 50	12/42	0.93		(0.42–2.09)	0.87	1.08	(0.47–2.50)	0.86
	50 ≤ PP < 64	26/83	0.95		(0.47–1.93)	0.9	1.04	(0.51–2.13)	0.91
	PP ≥ 64	45/124	1.06		(0.55–2.06)	0.86	1.16	(0.59–2.27)	0.66
Untreated (N = 946)	PP < 40*	34/290	Ref				Ref		
	40 ≤ PP < 50	58/249	1.67		(1.09–2.57)	0.019	1.52	(0.98–2.36)	0.058
	50 ≤ PP < 64	53/224	1.5		(0.96–2.34)	0.075	1.31	(0.84–2.06)	0.24
	PP ≥ 64	77/183	2.54		(1.65–3.91)	<0.001	2.34	(1.51–3.63)	< 0.001

Age was adjusted by considering it as the time-scale

Model 1: Adjusted for sex

Model 2: Adjusted for sex, BMI, diabetes, hypercholesterolemia, eGFR, smoking and anti-hypertensive medication (only for total population)

*BP* blood pressure, *SBP* systolic blood pressure, *DBP* diastolic blood pressure, *pp* pulse pressure, *E/N* events/N, *HR* hazard ratio, *CI* confidence interval

*Reference intervals

**Table 3:** Multivariate adjusted risk estimation of different systolic and diastolic blood pressure as well as pulse pressure quartiles for total mortality in total population and those with and without anti-hypertensive medication: Tehran Lipid and Glucose study (1999–2014)

	BP category		Model 1			Model 2		
		E/N	HR	(95% CI)	p	HR	(95% CI)	p
Total (N = 1448)	SBP < 120	41/413	1.01	(0.64–1.58)	0.96	1.05	(0.67–1.64)	0.84
	120 ≤ SBP < 130*	36/260	Ref			Ref		
	130 ≤ SBP < 140	60/253	1.69	(1.12–2.56)	0.013	1.40	(0.92–2.14)	0.11
	SBP ≥ 140	168/522	1.84	(1.28–2.64)	0.001	1.72	(1.19–2.48)	0.004
Treated (N = 399)	SBP < 120	12/52	1.55	(0.67–3.62)	0.31	1.79	(0.75–4.26)	0.19
	120 ≤ SBP < 130*	10/45	Ref			Ref		
	130 ≤ SBP < 140	20/70	1.71	(0.79–3.68)	0.17	1.66	(0.75–3.67)	0.21
	SBP ≥ 140	73/232	1.54	(0.79–2.99)	0.2	1.78	(0.9–3.53)	0.1
Untreated (N = 1049)	SBP < 120	29/361	0.92	(0.54–1.56)	0.75	0.92	(0.54–1.57)	0.77
	120 ≤ SBP < 130*	26/215	Ref			Ref		
	130 ≤ SBP < 140	40/183	1.64	(1.0–2.71)	0.049	1.34	(0.81–2.23)	0.25
	SBP ≥ 140	95/290	1.88	(1.21–2.91)	0.005	1.81	(1.16–2.83)	0.009
Total (N = 1448)	DBP < 80	136/640	1.79	(1.22–2.65)	0.003	1.68	(1.13–2.5)	0.01
	80 ≤ DBP < 85*	32/276	Ref			Ref		
	85 ≤ DBP < 90	40/194	1.85	(1.16–2.96)	0.009	1.78	(1.12–2.85)	0.015
	DBP ≥ 90	97/338	2.66	(1.78–3.98)	<0.001	2.77	(1.85–4.15)	<0.001
Treated (N = 399)	DBP < 80	43/120	2.92	(1.42–6.02)	0.004	2.73	1.3–5.7)	0.008
	80 ≤ DBP < 85*	9/76	Ref			Ref		
	85 ≤ DBP < 90	12/56	1.99	(0.84–4.74)	0.12	2.02	(0.84–4.88)	0.11
	DBP ≥ 90	51/147	3.68	(1.81–7.5)	<0.001	4.53	(2.21–9.3)	<0.001
Untreated (N = 1049)	DBP < 80	93/520	1.47	(0.93–2.33)	0.1	1.41	(0.88–2.26)	0.15
	80 ≤ DBP < 85*	23/200	Ref			Ref		
	85 ≤ DBP < 90	28/138	1.88	(1.08–3.27)	0.025	1.88	(1.07–3.28)	0.027
	DBP ≥ 90	46/191	2.25	(1.36–3.72)	0.001	2.17	(1.3–3.61)	0.003
Total (N = 1448)	PP < 39*	28/363	Ref			Ref		
	39 ≤ PP < 51	57/382	1.41	(0.9–2.23)	0.13	1.27	(0.8–2.02)	0.3
	51 ≤ PP < 65	91/346	1.79	(1.16–2.76)	0.008	1.51	(0.97–2.33)	0.06
	PP ≥ 65	129/357	1.94	(1.26–2.97)	0.002	1.71	(1.11–2.63)	0.014
Treated (N = 399)	PP < 49*	24/109	Ref			Ref		
	49 ≤ PP < 60	28/94	1.27	(0.72–2.22)	0.4	1.29	(0.73–2.28)	0.34
	60 ≤ PP < 74	33/99	1.03	(0.6–1.78)	0.9	0.91	(0.52–1.59)	0.76
	PP ≥ 74	30/97	0.88	(0.5–1.54)	0.65	0.84	(0.48–1.48)	0.54
Untreated (N = 1049)	PP < 39*	18/279	Ref			Ref		
	39 ≤ PP < 48	22/263	0.88	(0.47–1.64)	0.68	0.66	(0.35–1.26)	0.21
	48 ≤ PP < 61	56/256	1.7	(0.99–2.93)	0.05	1.22	(0.7–2.14)	0.48
	PP ≥ 61	94/251	2.18	(1.29–3.69)	0.003	1.83	(1.07–3.12)	0.027

Age was adjusted by considering it as the time-scale

Model 1: Adjusted for sex

Model 2: Adjusted for sex, BMI, prevalent CVD, diabetes, hypercholesterolemia, eGFR, smoking and anti-hypertensive medication (only for the total population)

*BP* blood pressure, *SBP* systolic blood pressure, *DBP* diastolic blood pressure, *pp* pulse pressure, *E/N* events/N, *HR* hazard ratio, *CI* confidence interval

*Reference intervals

In the total population, participants with DPB < 80 mmHg had higher HR, compared to the reference group. When the analysis was stratified by treatment group, a positive but statistically non-significant risk also was observed for both treated and untreated groups ([2.0 (0.89–4.54)] and [1.43 (0.95–2.16)] respectively. Comparing different quartiles of PP for CVD events, the highest risk was seen in participants with PP ≥ 64 mmHg in the total population as well as among the untreated group (Table 2).

Studying all-cause mortality, in multivariate analysis, the highest HR was noted in SBP ≥ 140 mmHg among the total population as well as untreated participants. Comparing the four different DBP groups, among those with DBP ≥ 85 mmHg (whether DBP 85–90 or ≥ 90 mmHg) there was an increased risk of all-cause mortality in the total population, as well as the untreated group. HR was highest among participants with DBP > 90 mmHg in the total population as well as treated and untreated groups. Besides, pooling DBP 85–90 and ≥ 90 mmHg as a single group, DBP ≥ 85 showed significant risk for total mortality among the total as well as treated and untreated populations; the corresponding multivariate adjusted HR (CI) were 2.38 (1.62–3.51), 3.7 (1.82–7.5) and 2.04 (1.27–3.3), respectively. Furthermore participants with DBP < 80 mmHg had higher HR, compared with the reference group, in the total population and the treated group. Focusing on PP, the highest statistically significant HR for all-cause mortality was seen in those with PP ≥ 65 mmHg in the total population and those with PP ≥ 61 mmHg in the untreated group. In the treated group results were not statistically significant (Table 3).

The multivariate adjusted risk estimation of different SBP, DBP, as well as PP quartiles for CV-mortality in the total population, is shown in Table 4. Hazard ratio for CV mortality events in the total population was highest among participants with SBP ≥ 140 mmHg. Regarding DBP, not only HR was increased in participants with DBP ≥ 85 mmHg, (i.e. DBP 85–90 or ≥ 90 mmHg), but a statistically significant increased HR was also seen in DBP < 80 mmHg, compared with the reference group. Pooling DBP 85–90 and ≥ 90 mmHg as a single group, DBP ≥ 85 mmHg showed a multivariate adjusted HR [6.3 (2.5–15.9)] for CV mortality in the total population. Participants with PP ≥ 64 mmHg had highest HR, compared with the reference quartile of PP. Due to the low incidence for CV-mortality, we could not analyze the treated and untreated groups separately.

**Table 4:** Multivariate adjusted risk estimation of different systolic and diastolic blood pressure and pulse pressure quartiles for CV-mortality in the total population and those with and without anti-hypertensive medication: Tehran Lipid and Glucose study (1999–2014)

	BP category		Model 1			Model 2		
		E/N	HR	(95% CI)	p	HR	(95% CI)	p
Total (N = 1232)	SBP < 120	14/367	1.03	(0.47–2.23)	0.95	1.11	(0.51–2.42)	0.79
	120 ≤ SBP < 130*	12/224	Ref			Ref		
	130 ≤ SBP < 140	13/211	1.14	(0.52–2.49)	0.75	0.92	(0.41–2.05)	0.85
	SBP ≥ 140	61/430	2.07	(1.11–3.86)	0.02	2.21	(1.16–4.22)	0.016
	DBP < 80	43/544	3.69	(1.46–9.34)	0.006	3.0	(1.17–7.7)	0.022
	80 ≤ DBP < 85*	5/242	Ref			Ref		
	85 ≤ DBP < 90	17/165	5.13	(1.89–13.9)	0.001	5.48	(2.01–14.92)	0.001
	DBP ≥ 90	35/281	6.36	(2.48–16.32)	< 0.001	6.86	(2.66–17.7)	< 0.001
	PP < 40*	9/327	Ref			Ref		
	40 ≤ PP < 50	19/291	1.69	(0.76–3.76)	0.19	1.58	(0.7–3.54)	0.27
	50 ≤ PP < 64	23/307	1.44	(0.68–3.27)	0.31	1.25	(0.57–2.75)	0.58
	PP ≥ 64	49/307	2.49	(1.20–5.20)	0.014	2.22	(1.06–4.64)	0.034

Subjects with prevalent CVD were excluded for CV-mortality

Model 1: Adjusted for sex

Model 2: Adjusted for sex, BMI, diabetes, hypercholesterolemia, eGFR, smoking and anti-hypertensive medication (only for the total population). Age was adjusted by considering it as the time-scale

*CVD* cardiovascular disease, *BP* blood pressure, *SBP* systolic blood pressure, *DBP* diastolic blood pressure, *pp* pulse pressure, *E/N* events/N, *HR* hazard ratio, *CI* confidence interval

*Reference intervals

**Fig. 1:**
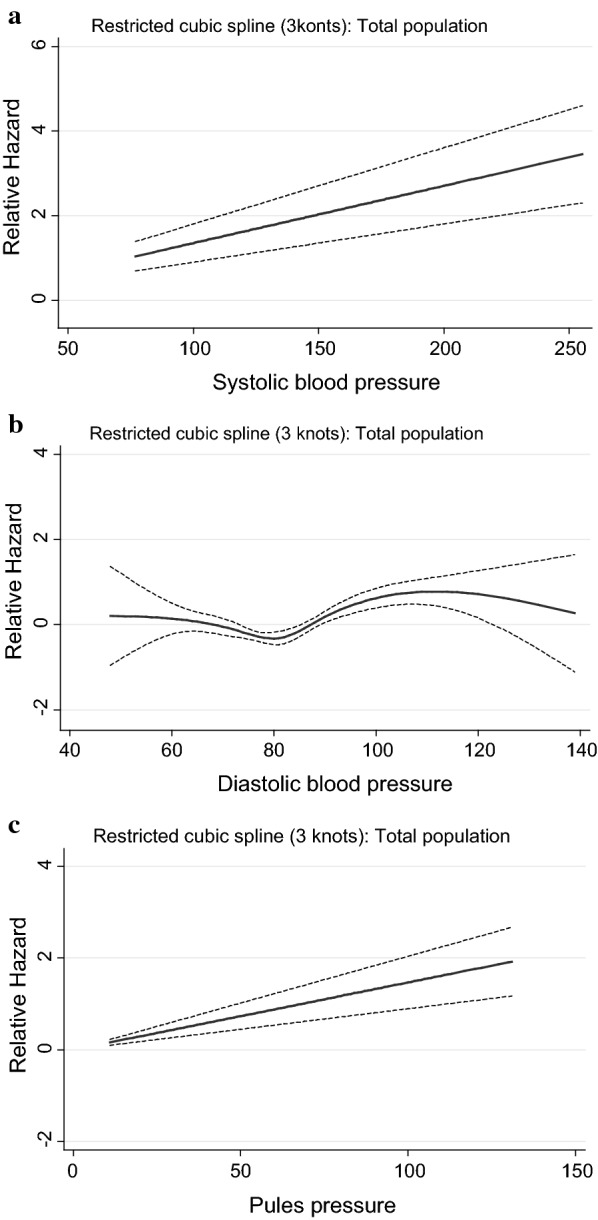
Dose–response relationship between risk of CVD events with systolic blood pressure, diastolic blood pressure and pulse pressure as continuous variables, in the total population. The relationship was linear for SBP (**a**), U-shaped for DBP (**b**) and linear for PP (**c**)

**Fig. 2:**
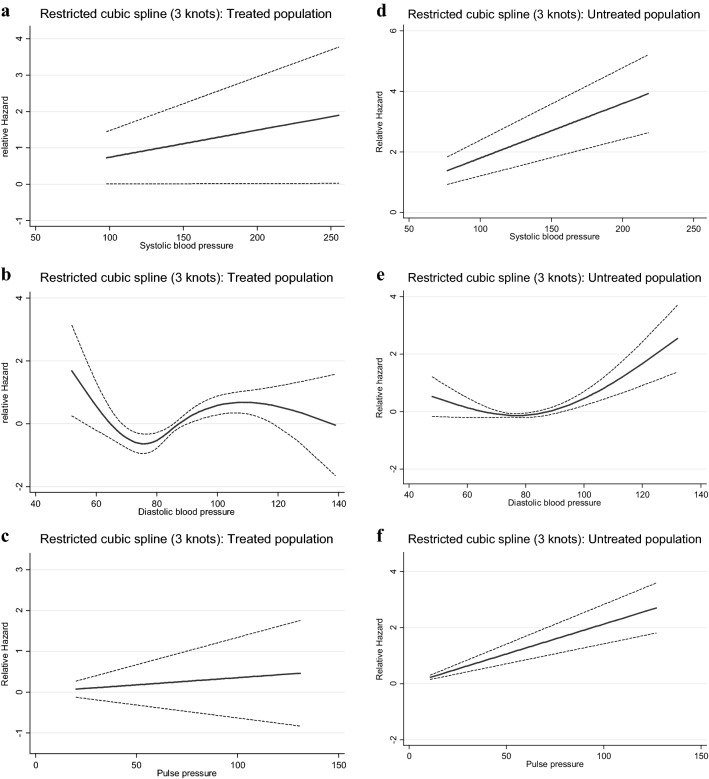
Dose–response relationship between risk of CVD events with systolic blood pressure, diastolic blood pressure as well as pulse pressure as continuous variables in the treated and untreated populations. In the treated population, the relationship was linear for SBP (**a**), U-shaped for DBP (**b**) and linear for PP (**c**). In the untreated population the relationship was linear for SBP (**d**), U-shaped for DBP (**e**) and linear for PP (**f**)

**Fig. 3:**
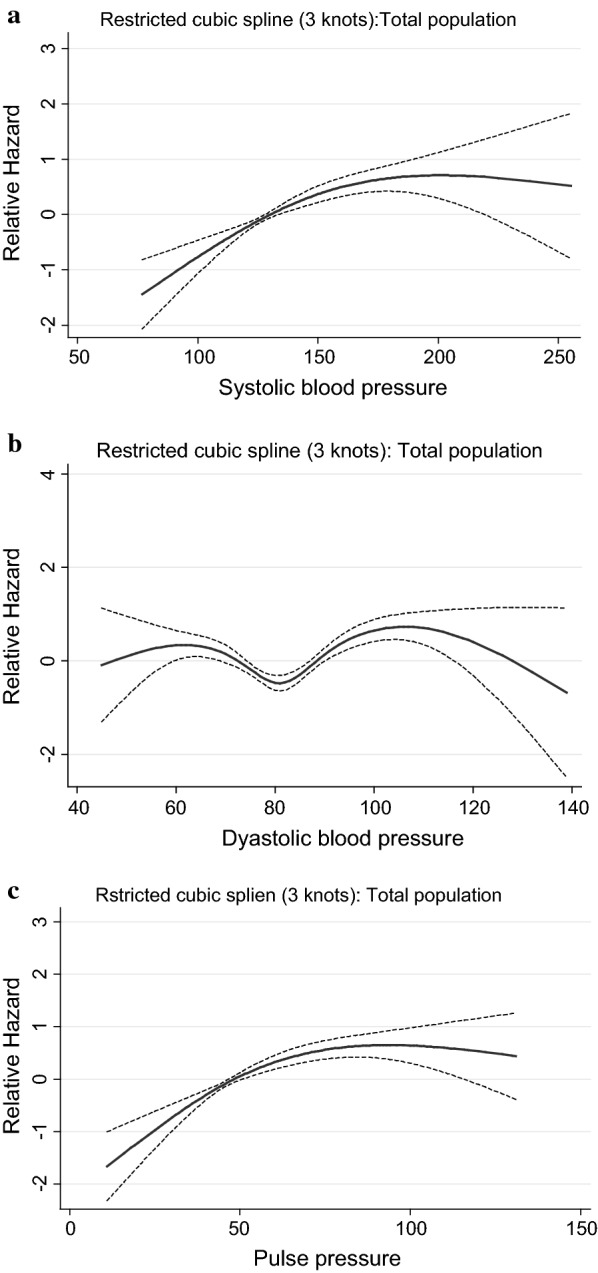
Dose–response relationship between risk of total mortality with systolic blood pressure, diastolic blood pressure and pulse pressure as continuous variables in the total population. The relationship was non-linear for SBP (**a**), U-shaped for DBP (**b**) and non-linear for PP (**c**)

**Fig. 4:**
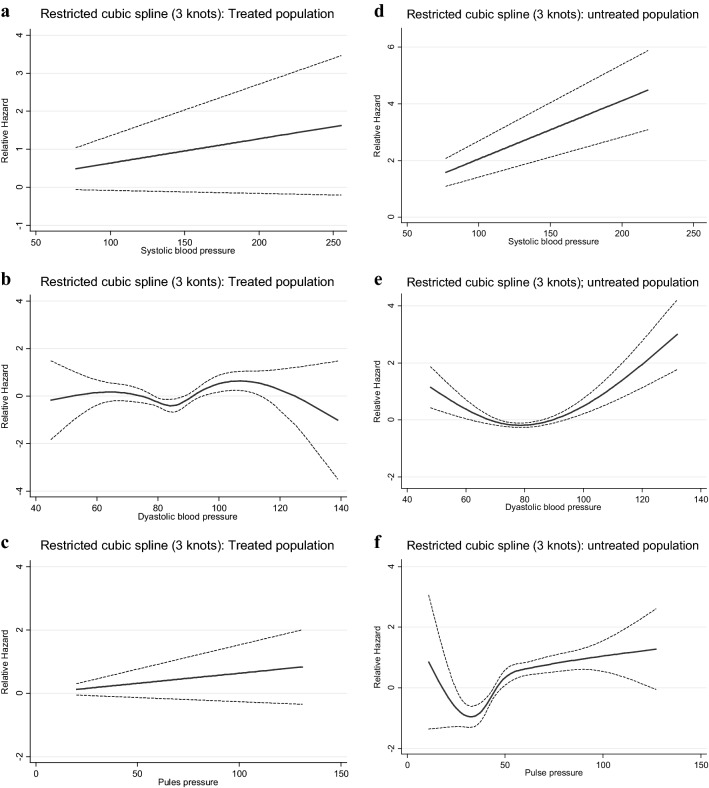
Dose–response relationship between risk of total mortality with systolic blood pressure, diastolic blood pressure and pulse pressure as continuous variables, in the treated and untreated population. In the treated population, the relationship was linear for SBP (**a**), U-shaped for DBP (**b**) and linear for PP (**c**). In the untreated population, the relationship was linear for SBP (**d**), U-shaped for DBP (**e**) and non-linear for PP (**f**)

**Fig. 5:**
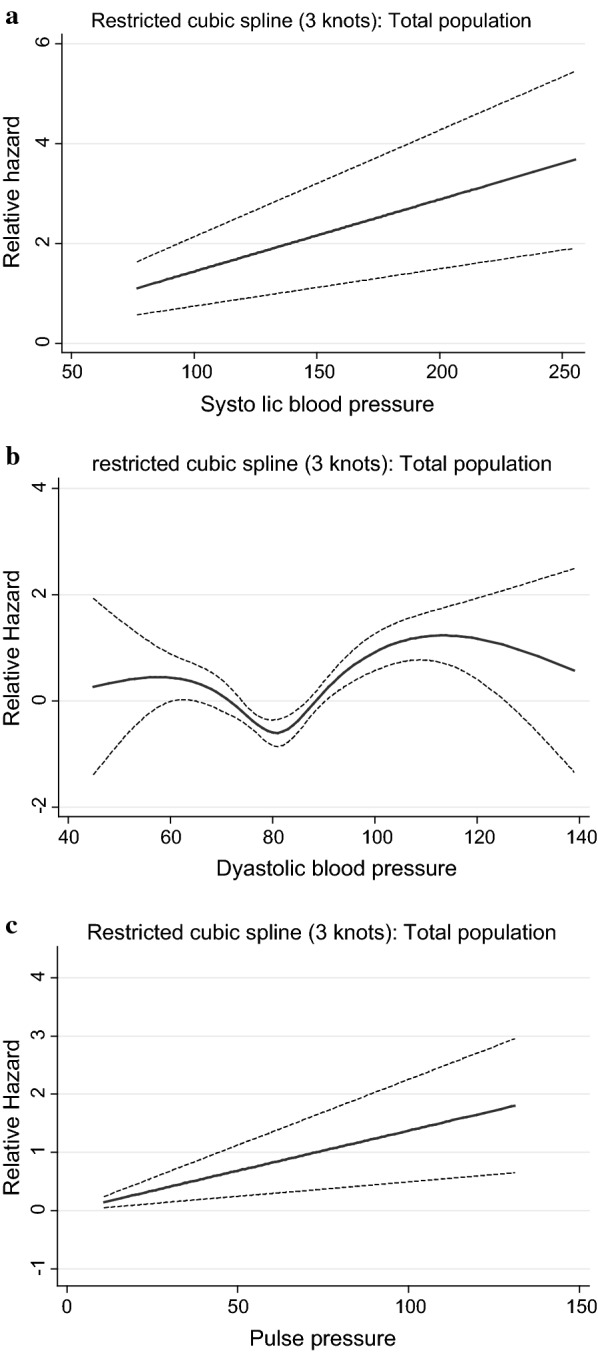
Dose–response relationship between risk of CV-mortality with systolic blood pressure, diastolic blood pressure and pulse pressure as continuous variables, in the total population. The relationship was linear for SBP (**a**), U-shaped for DBP (**b**) and linear for PP (**c**)

Figures 1, 2, 3, 4 and 5 show, the dose–response relationship between SBP, DBP and PP with the outcomes under the investigation. A linear relationship was shown between SBP and PP with CVD events, among the total population (Fig. 1) as well as among treated and untreated groups (Fig. 2). Considering total mortality among the total population (Fig. 3), neither SBP nor PP showed a linear relationship. However, when we stratified by treatment (Fig. 4), for SBP the relationship was linear in both the treated and untreated groups, whereas for PP, the linear relationship was only found among the treated group. For CV mortality in the total population (Fig. 5), SBP and PP showed a linear relationship; For DBP there was a U-shaped relationship with CVD events, all-cause and CV-mortality after multivariable adjustment. Relationships between DBP with CVD events and total mortality also showed a U-shaped pattern when results were stratified by treatment groups. Due to the low incidence for CV mortality, we could not analyze the treated and untreated groups separately.

## Discussion

Over a decade long follow-up among CKD patients in a population based study, we explored the association between SBP, DBP and PP with CVD and mortality events. Our results revealed a generally linear association between SBP with CVD and mortality events. In multivariate analysis, comparing those with 120 ≤ SBP < 130 mmHg as the reference, those with SBP ≥ 140 mmHg, showed over 60% increased risk for both CVD and all-cause mortality events, and an over twofold risk for CV-mortality. Considering DBP, a U-shaped relationship with CVD and mortality events was found. In multivariate analysis, with 80 ≤ DBP < 85 mmHg as the reference, patients with DBP < 80 or DBP ≥ 85 mmHg both showed a significantly higher positive risk for events; more importantly, the risk reached over 200% for CV mortality in patients with DBP < 80 mmHg. The U shaped association between DBP and events was also evident in the hypertensive-treated group, given that the risk for all-cause mortality events in DBP < 80 mmHg exceeded over 170%. Hence, based on results of this observational study, SBP < 140 and 80 ≤ DBP < 85 mmHg were associated with the lowest risk for CVD and mortality events. Similar to SBP, generally a linear association was demonstrated between PP with CVD and mortality events. In fact patients in the 4th quartile of PP had an over 70% risk for both CVD and all-cause mortality, in comparison to the reference group.

The associations of different components of blood pressure with CVD and mortality events among CKD patients has been addressed in several studies, however, to the best of our knowledge no study has examined the impact of all three main BP components (SBP, DBP, PP) on CVD and mortality events in a single study.

The increased risk of events we observed in SBP ≥ 140 group is consistent with the SBP goal of JNC 8 [5]; meanwhile although not statistically significant, for all-cause mortality, the increased risk was evident in those with SBP more than 130, results more in line with the new AHA recommendations of reducing SBP to below 130 mmHg for CKD patients [6]. The pattern we observed between SBP and outcomes echoes results of the SPIRINT randomized controlled trials [8, 9] and those of Bansal et al. [7]; “*The lower the better strategy*” was supported by results of the SPIRINT study, demonstrating lower rates of adverse events for SBP below 120 mmHg, in comparison to SBP below 140 mmHg in both CKD and non-CKD patients [8, 9]. Bansal et al. [7], in an observational study conducted on 1795 advanced CKD patients (stages 4 and 5), linked a higher rate of atherosclerotic cardiovascular events (ASCVD) to higher SBPs with a linear pattern. Relationships of DBP and PP with ASCVD were also reported as linear in this American population based study [7]. However, there are observational studies among CKD patients that have reported a U shape association between SBP and all-cause mortality events [10, 12]. Kovesdy et al. [10], among mostly elderly men with CKD, mean age around 74 year, found that SBP < 130 mmHg or ≥ 160 mmHg was associated with higher mortality events, regardless of accompanying DBP. Additionally, Weiss et al. [12], found different relationships between SBP and all-cause mortality in different age groups among elder adults, aged ≥ 65 years with CKD; they found a U shaped pattern among participants, aged 65–70, but for those ≥ 70 year. higher mortality was linked with lower values of SBP. Interestingly, in our study only among hypertensive treated patients with CKD, SBP below 120 was associated with approximately 40 and 80% increase in risk for CVD and all-cause mortality events, respectively, neither of which were statistically significant, probably due to limited number of events. The difference observed in the association between SBP and outcomes might be attributable to the younger age of our study population (mean age of 60.9 year), compared with these two population based studies from the US [10, 12].

The U shaped pattern we found regarding the relationship between DBP and all-cause mortality in CKD patients supports the results of Kovesdy et al. [10, 13]. More importantly we also showed the same U shaped pattern even with higher HRs in the hypertensive-treated group compared to the untreated group (HR 2.73 and 1.41 respectively), suggesting that DBP < 80 mmHg may even cause harm to CKD patients. In other words, our results suggest that in CKD patients lowering SBP at the expense of lowering DBP to below 80 mmHg can potentially increase morbidity and mortality rates. The higher CVD and mortality events observed in patients with low DBP can be explained by several theories: First, as most of the coronary blood flow occurs during diastole, patients with low DBP may be more susceptible to CVD events [27]. Second, patients with underlying chronic disease such as neoplasms, chronic infection, malnutrition and heart failure have lower DBP, indicating preexisting poor health status and residual confounding, lead to higher CVD and mortality events among the low DBP group, a phenomena called “reverse causality” [27, 28]. To address this concern, we omitted the mortality events during first 3 years of our follow-up; however the U shaped association between DBP and events remained essentially unchanged (data not shown). Third, some studies showed unintentionally reducing eGFR by tight blood pressure regimens, is itself an independent risk factor for CVD [27, 29].

The complex interplay of the different BP components described above, adds to the dilemma of BP control in CKD patients, as there are individuals with high SBP but normal or even below normal DBP in this population. With antihypertensive therapy, these patients will be at risk of low DBP, at some point during their course of treatment. This suggests further investigations to look for an appropriate “combination range of SBP and DBP” for optimal BP control in CKD patients.

Considering PP, our results are similar to the results of Palit [14] and Bansal [7] showing higher rates of events with higher PP in a linear pattern. CKD patients are more prone to have higher PPs and the average PP in our study was 52.8 mmHg, a level which was lower than those of the Palit [14] and Bansal [7] studies, both of which were conducted on advanced CKD patients. The extra damage to the vascular wall in addition to increased stress on the left ventricle wall are two possible explanations of the higher morbidity and mortality observed in CKD patients with higher PP [30, 31]. Large artery stiffness due to advanced atherosclerosis and accelerated medial calcification seen in CKD patients [14], makes SBP more resistant to BP lowering therapy, often necessitating extra medication to achieve SBP goals. On the other hand, poor vascular compliance in CKD patients can increase susceptibility to diastolic hypotension. Hence intensive blood pressure control regimens can further exacerbate wide ranges of PP and its related risks in CKD patients [32].

One of the interesting findings in our study is the fact that the survivor group had higher baseline values of BMI, total cholesterol and number of patients with hypercholesterolemia compared to those who died. Some but not all studies conducted among CKD patients, interpreted similar findings by stating that higher BMI might be an index of better overall health status, less frailty and or less muscle wasting, a phenomena called “the obesity paradox” [33]. The disparity among evidence on this issue may be related to differences in study populations, length of follow-up, covariate adjustment, and/or investigated outcomes [34]. Furthermore, relationships between elevated BMI and ESRD or mortality may be weaker in cohorts of individuals with CKD, which may be related to the increased risk of muscle wasting (i.e. frailty) in this population [35] and limitations of BMI in distinguishing body composition or fat distribution [36].

There are number of limitations to our study. First, due to the observational nature of this study we cannot establish a cause-and-effect relationship between different BP components and outcomes, regarding unmeasured probable confounders. Second, due to the limited number of events we did not analyze the effect of the three different components of BP in the treated versus untreated subgroups separately for CV mortality. Third, we did not have data about urinary albumin excretion, hence albuminuria in not considered in the CKD definition. Fourth, the average eGFR in our CKD population is rather high (52.8 ml/min per 1.73 m^2^) and as a result, our findings might not be extrapolated to patients with more advanced renal failure. Fifth, using the MONICA protocol in TLGS cohort, the BP measurements are performed only from right arm, hence interarm blood pressure discrepancy (IAD) was not assessed in our study. Nevertheless in the general population, IAD levels > 20 mmHg, usually associated with vascular disease and its related adverse outcomes, are quite infrequent, occurring in less than 4% of population [37]. Lastly, the study was conducted only among a Tehranian population; and therefore results might not be generalized to other parts of the country.

## Conclusions

This is the first cohort study of CKD patients in a Middle Eastern population, with more than a decade long follow-up, which examines the effect of all the three different BP components for CVD and mortality events. According to our findings, maintaining SBP at levels < 140 mmHg, DBP between 80 and 85 mmHg and PP < 64 mmHg were associated with lowest risk for CV and all-cause mortality events.

## Abbreviations

CVD: cardiovascular disease
CV-mortality: cardiovascular mortality
CKD: chronic kidney disease
BP: blood pressure
JNC: Joint National Committee
AHA: American Heart Association
ACC: American College of Cardiology
SBP: systolic blood pressure
DBP: diastolic blood pressure
PP: pulse pressure
TLGS: Tehran lipid and glucose study
FPG: fasting plasma glucose
2 h-PCG: 2-h post challenge plasma glucose
TC: total cholesterol
BMI: body mass index
eGFR: estimated glomerular filtration rate
Cr: creatinine
ICD: International Classification of Diseases
CKD-EPI: chronic kidney disease epidemiology collaboration formula
T2D: type 2 diabetes
HR: hazard ratio
SD: standard deviation
ESRD: end-stage renal disease
